# Chemical characterization of baby food consumed in Italy

**DOI:** 10.1371/journal.pone.0297158

**Published:** 2024-02-22

**Authors:** Maria Assunta Meli, Donatella Desideri, Davide Sisti, Ivan Fagiolino, Carla Roselli

**Affiliations:** 1 Department of Biomolecular Sciences, Università di Urbino Carlo Bo, Urbino (PU) Italy; 2 GRUPPO C.S.A. S.p.A., Rimini (RN) Italy; University of Alcala Faculty of Medicine and Health Sciences: Universidad de Alcala Facultad de Medicina y Ciencias de la Salud, SPAIN

## Abstract

In this study, a total of 30 elements (essential and non-essential or toxic) were determined in 25 foods consumed in Italy by children aged 0–6 months and produced in Europe. Inductively Coupled Plasma-Atomic Emission Spectrometry and Inductively Coupled Plasma-Mass Spectrometry were used as measurement techniques for the elements of interest. The estimated intakes for one-year-old infants were compared with risk estimators and nutritional requirements. Data indicate that commercially available baby food in Italy provides an excellent contribution for Mn, Cu, Fe, Zn, Ca, K, and P, covering up to approximately 70% of the adequate intake (AI) for an infant aged 6–12 months. The intake of detectable toxic elements was always below the safety limit: even the most concentrated toxic elements never exceeded about 86% of the Provisional Tolerable Weekly Intake (PTWI). This result indicates that the analyzed baby food is of good quality and does not pose risks to children’s health.

## Introduction

The period from birth to 12 months of age represents a critical phase for cognitive and emotional development as well as rapid physical growth in children. Ensuring proper nutrition during this period is essential to support adequate psycho-physical development [1].

While breast milk remains the optimal food for newborns due to its richness in immune defense proteins, powdered milk fully meets the nutritional needs [2]. However, according to a survey conducted by the Italian National Institute of Health between December 2018 and April 2019, involving 29,492 mothers across 11 Italian Regions, findings revealed that less than one quarter (23.6%) of children at 4–5 months were exclusively breastfed, and 11.7% of children in the monitored age group had not been breastfed at all. Considering the widespread use of infant foods in Italy and other industrialized countries, verifying the actual quality of these products is highly important [3].

Physiological changes occurring in infants during the first six months enable them to transition from a solely liquid diet to solid foods, mainly comprising homogenized meat, vegetables, cheese, fruits, and cereal flours. Initial complementary foods include rice cereal and cereal cream, followed by the introduction of fruits and vegetables between 6 and 8 months. By 8 to 12 months, soft meat can also be introduced, highlighting the significance of assessing adequate nutrient intake and identifying potential contaminants during this developmental stage.

The presence of elements considered toxic (As, Cd, Hg, Pb, Sb) in the ecosystem, exacerbated by anthropogenic activities in recent decades [4, 5], raises concerns about environmental and food contamination. As food serves as the primary route of internal contamination, ensuring the quality control of food contaminants becomes crucial, particularly for sensitive groups like children [6, 7]. Rapid development of children’s nervous, reproductive, digestive, and respiratory systems makes them more susceptible to potential risks from toxic elements, necessitating careful monitoring [8–12].

Essential elements’ adequate intake is crucial for optimal bodily functions, as their deficiency can impact vital functions, while excessive intake over prolonged periods can lead to adverse effects [13]. Essential metals like Fe, Co, Cu, Mn, and Zn, although necessary, may exhibit toxic properties at higher concentrations [14], affecting enzyme metabolism and influencing susceptibility to various viral infections [4]. While essential, chromium (Cr) can exist in carcinogenic hexavalent forms [15], potentially affecting overall health.

The presence of contaminants in baby food poses health risks, especially considering the rapid organ development during the first year of life, categorized into functional systems [2]. Infants, being the most vulnerable group, are highly sensitive to exposure due to increased intestinal absorption and lower thresholds for adverse effects. Hence, determining the concentration of significant toxic elements in the common diet becomes crucial to assess infants’ exposure and levels of essential macro and micronutrients necessary for optimum growth.

This study conducted measurements to determine the levels of 30 elements, categorized into essential, major (K, Ca, Mg, P, S), minor or trace (Mn, Fe, Cu, Zn, Co, Cr, and Si), and non-essential or toxic (Al, Ba, Rb, Sr, As, Cd, Sn, Ce, La, Tl, Te, Ti, Th, U, Hg, Sb, Ni, and Pb) in various baby foods available in large-scale retail trade in Italy. The study aimed to: a) assess the suitability of the 25 analyzed baby foods for infant consumption by estimating the intake of toxic elements and comparing it with safety limits set by the Joint FAO/WHO Expert Committee on Food Additives (JECFA) [16–20]; b) evaluate the benefits of baby food consumption by assessing the intake of essential elements and comparing it with nutritional requirements established by EFSA’s Population Reference Intake (PRI) and Adequate Intake (AI) [21]; c) build upon previous research by the authors on overall dietary patterns [4, 5, 22] and specifically infant diet [23].

## Materials and methods

### Sampling and sample preparation

These samples were purchased from major supermarket chains. Some of them indicated the manufacturing country (Italy, Germany, Spain, Holland, Switzerland), while others were labeled only as ’produced in the E.U.’ (Table 1). The food items were categorized into three groups: cream of rice [13], homogenized (3 fruits, 3 fish, 4 meat, 2 baby cheese), and powdered milk [14], which serves as the primary food for infants in the first 6 months, substituting human breast milk. Regarding the homogenized samples, approximately 100 g was frozen at -20°C for 12 hours and then freeze-dried for 24 hours using the EDWARDS dryer module. The dehydrated samples were weighed, and the average ratio percent between dry and wet weight (dw/ww) was 18.3 ± 3.30%.

**Table 1:** Baby food analysed, manufacturing country and, for powdered milk, metals concentration (mg kg^-1^) reported in the labels by manufactures, mean and standard deviation (SD).

Food	Identification Number	Manufacturing Country	K	Ca	Mg	P	Fe	Cu	Zn	Mn
*Powder milk*	1	Italy	4440	4810	330	3330	60	2.9	31	0.85
	2	Germany	6960	4210	490	3260	32	2.8	38	0.96
	4	Germany	5000	4800	460	3200	49	2.5	43	0.59
	5	Switzerland	4800	4500	400	3000	59	2.9	59	0.30
	6	European Union	4779	3382	375	1912	39	2.9	37	0.55
	7	European Union	4779	3382	375	1912	39	2.9	37	0.55
	8	Germany	5300	5050	450	2850	46	3.0	51	0.51
	9	Germany	5850	4300	400	2400	46	2.9	52	0.51
	10	Holland	5250	3300	440	1830	52	4.0	54	1.36
	*Mean*		*5480*	*3480*	*340*	*2460*	*60*	*3*.*0*	*30*	*0*.*33*
	*SD*		*5264*	*4122*	*406*	*2615*	*48*	*3*.*0*	*43*	*0*.*65*
*Homogenized food*										
rabbit	11	European Union								
lamb	12	European Union								
turkey	13	Spain								
veal	14	European Union								
bream	15	Italy								
salmon	16	Italy								
seabass	17	Italy								
cheese	18	European Union								
cheese	19	Italy								
apple	20	Italy								
pear	21	Italy								
banana	22	Italy								
*Cereal cream*	23	European Union								
	24	European Union								
	25	European Union								

### Elemental analysis

#### Sample dissolution

The sample dissolution was conducted in accordance with EPA 3050B 1996 method [24]. A dry weight sample of 1–1.5 g was digested in a mixture of 3 ml concentrated nitric acid, 10 ml concentrated hydrochloric acid and 1 ml 30% hydrogen peroxide. All steps were described in detail by Meli et al. [4].

#### Measure methods

After dissolving the samples, three different methods were employed for elemental analysis: ICP-AES, ICP-MS, and AAS for Hg [25, 26]. The broad spectrum of analytical applications, extended dynamic concentration ranges, high selectivity and sensitivity, along with low analytical limits, establish ICP-AES and ICP-MS as the preferred instrumental methods for determining trace elements in various samples. The limits of detection (LOD) (mg kg^-1^_ww_) are outlined in Table 2.

**Table 2:** Elements determined in baby food and relevant method of determination, limit of detection, LOD (mg kg-1ww) (number of the sample with concentration > LOD) and limit of quantification, LOQ.

	Element	LOD	LOQ	Method
Essential (Minor and trace)	Calcium	500 (17)	1500	EPA 3050B 1996 + EPA6010D 2014
	Chrom (total)	0.50 (0)	1.50	EPA 3050B 1996 + EPA6010D 2014
	Cobalt	0.50 (0)	1.50	EPA 3050B 1996 + EPA6010D 2014
	Copper	0.50 (23)	1.50	EPA 3050B 1996 + EPA6010D 2014
	Iron	2.50 (25)	7.50	EPA 3050B 1996 + EPA6010D 2014
	Magnesium	0.50 (25)	1.50	EPA 3050B 1996 + EPA6010D 2014
	Manganese	0.50 (25)	1.50	EPA 3050B 1996 + EPA6010D 2014
	Phosphorus	0.50 (25)	1.50	EPA 3050B 1996 + EPA6010D 2014
	Potassium	500 (25)	1500	EPA 3050B 1996 + EPA6010D 2014
	Silicium	10.0(18)	30.0	EPA 3050B 1996 + EPA6010D 2014
	Sulphur	1.00 (25)	3.00	EPA 3050B 1996 + EPA6010D 2014
	Zinc	0.50 (25)	1.50	EPA 3050B 1996 + EPA6010D 2014
Non essential or toxic	Aluminium	2.50 (9)	7.50	EPA 3050B 1996 + EPA6010D 2014
	Antimony	0.10 (25)	0.30	EPA 3050B 1996 + EPA6010D 2014
	Arsenic	0.10 (3)	0.310	EPA 3050B 1996 + EPA6010D 2014
	Barium	2.50 (0)	7.50	EPA 3050B 1996 + EPA6010D 2014
	Cadmium	0.005 (0)	0.015	EPA 3050B 1996 + EPA6010D 2014
	Cerium	1.00 (0)	3.00	EPA 3050B 1996 + EPA6020D 2014
	Lantanium	1.00 (0)	3.00	EPA 3050B 1996 + EPA6020D 2014
	Lead	0.10 (0)	0.30	EPA 3050B 1996 + EPA6010D 2014
	Mercury	0.0005 (25)	0.0015	EPA 3050B 1996 + EPA 7473 2007
	Nickel	0.50 (4)	1.50	EPA 3050B 1996 + EPA6010D 2014
	Rubidium	1.00 (0)	3.00	EPA 3050B 1996 + EPA6020D 2014
	Strontium	0.50(24)	1.50	EPA 3050B 1996 + EPA6010D 2014
	Tallium	0.10 (0)	0.30	EPA 3050B 1996 + EPA6010D 2014
	Tellurium	0.50 (0)	1.50	EPA 3050B 1996 + EPA6010D 2014
	Tin	0.50(17)	3.00	EPA 3050B 1996 + EPA6010D 2014
	Titanium	0.50 (0)	1.50	EPA 3050B 1996 + EPA6010D 2014
	Thorium	1.00(0)	3.00	EPA 3050B 1996 + EPA6010D 2014
	Uranium	1.00 (0)	3.00	EPA 3050B 1996 + EPA6020D 2014

Mercury was identified using EPA 7470 1994, employing thermal decomposition, amalgamation, and atomic absorption spectrometry [27]. The LOD for mercury was 0.0001 mg kg^-1^_ww_.

**Quality control** measures were taken into consideration to address potential impurities from reagents and container release. A blank sample was prepared by mixing all reagents and employing the same procedures without the addition to the samples. Interferences were assessed, and necessary corrections were applied or data were flagged to indicate potential issues.

The accuracy of the method was validated through recovery tests with a Laboratory Control System (LCS), comprised of a blank sample supplemented with known quantities of analytes (reference sample). The average results obtained from replicates in the preparation and analysis of the reference sample deviated by 20% from the expected values (analytical standard errors). The reproducibility of metal determinations (precision), based on the variance in replicated analyses of the same sample, was lower by 10%.

*Data treatment*. The element concentrations for each sample were reported. Data was also grouped according to the samples classes and for each group the element concentration, expressed as an arithmetical mean and relative standard deviation (SD) are provided. In order to highlight correlations among elements concentrations, corrplot, using Pearson coefficients (r), were also shown. In corrplot, eccentricity of each ellipse quantify the strength of correlations between variables. Additionally, hierarchical clustering was performed to identify groups of elements showing similar covariations.

The nutritional significance of essential elements was evaluated based on recommended nutrient intakes, such as Population Reference Intakes (PRIs) and Adequate Intakes (AIs) [19]. Concurrently, the health hazards posed by toxic elements present in these products were assessed using risk estimators (PTWI) established by international scientific committees like the Joint FAO/WHO Expert Committee on Food Additives (JECFA) [2, 15–18].

The estimated daily intake of elements (EDI, mg kg^-1^ day^-1^) was computed using the formula:

EDI=C·I/B

Where C (mg kg^-1^) signifies the element concentration, I (I = kg day^-1^) denotes the daily ingestion rate, and B represents the body weight (kg). EFSA [21] suggested a reference body weight of 8.6 kg for an infant (male or female) aged 7–11 months. In this study, the ingestion rate reported for infants by UNSCEAR [28] was utilized. Concerning milk consumption, UNSCEAR recommends a consumption rate of 120 kg per year, equivalent to approximately 15.6 kg per year of powdered milk. This calculation assumes that approximately 13 kg of powdered milk can be produced from 100 L of fresh milk [29, 30], aligning with the ingestion rate of powdered milk recommended by ICRP for infants aged 6–12 months [31], and consistent with the manufacturer’s reconstitution instructions stated on the milk sample labels.

To facilitate a meaningful comparison between the estimated daily intake (EDI) and safety limits, all values were standardized into daily units.

## Results

### Essential and toxic elements concentration

Thirteen elements (Cr, Co, Ba, Cd, Ce, La, Pb, Rb, Tl, Te, Ti, Th, and U) exhibited concentrations below the Limit of Detection (LOD) in all samples (Table 2). Hence, to enhance sample characterization significantly, future efforts should focus on reducing detection limits using sample concentrating techniques. Tables 3 and 5 present the concentration (average of three replicates) of essential (Ca, Cu, Fe, K, Mg, Mn, P, S, Si, and Zn) and toxic elements (Al, As, Hg, Ni, Sb, Sn, and Sr) for each sample, along with the arithmetical mean and relative standard deviation (SD) for each group of baby food.

**Table 3:** Concentration (mg kg-1ww) of essential (major and minor or trace) elements in baby food, minimum, maximum value, mean, standard deviation (SD).

Food	Number	K	Ca	Mg	P	S	Fe	Mn	Cu	Zn	Si
*Powder milk*	1	4793	3729	256	2672	1075	53.7	0.50	2.30	32.0	<10
	2	7403	3777	388	2878	1162	28.5	1.00	2.30	41.8	16
	3	5570	4212	391	2999	1269	46.6	0.70	2.00	46.9	<10
	4	5466	4244	323	2753	1377	51.0	0.80	2.20	68.3	12
	5	5995	4265	372	2223	1114	43.7	0.50	3.60	42.3	<10
	6	5358	3432	307	1771	1213	37.5	0.60	2.20	40.6	<10
	7	5573	4301	377	2410	1231	50.4	0.80	2.60	53.2	<10
	8	7001	4597	415	2701	1129	50.9	0.50	3.20	55.1	12
	9	5190	3042	344	1585	1275	45.6	1.20	2.90	56.8	27
	10	6160	3105	253	2414	1304	57.1	0.60	2.40	34.3	<10
	*Mean*	*5851*	*3870*	*343*	*2441*	*1215*	*46*.*5*	*0*.*72*	*2*.*57*	*47*.*1*	*12*.*7*
	*SD*	*813*.*0*	*539*	*56*.*6*	*466*	*94*.*8*	*8*.*38*	*0*.*23*	*0*.*51*	*11*.*2*	*5*.*40*
*Homogenized food*											
rabbit	11	1173	<92.5	642	64	503	3.37	0.11	0.14	5.58	5.04
lamb	12	1168	<92.5	597	56	499	9.01	0.10	0.27	13.7	3.99
turkey	13	1281	<92.5	701	63	721	3.44	0.13	0.26	11.4	3.68
veal	14	893.0	<92.5	477	42	409	4.67	0.18	0.21	12.5	3.20
	*Mean*	*1129*	*<92*.*5*	*604*	*56*	*533*	*5*.*12*	*0*.*13*	*0*.*22*	*10*.*8*	*3*.*98*
	*SD*	*166*.*0*	*-*	*95*.*0*	*10*	*133*	*2*.*66*	*0*.*040*	*0*.*060*	*3*.*60*	*0*.*78*
bream	15	1005	84.9	415	65	322	1.22	0.29	0.27	1.43	7.20
salmon	16	1062	<92.5	449	69	337	1.43	0.39	0.23	1.50	7.50
sea bass	17	1070	100.7	401	75	366	3.57	0.54	0.23	1.59	7.65
	*Mean*	*1046*	*92*.*7*	*422*	*69*	*342*	*2*.*07*	*0*.*41*	*0*.*24*	*1*.*51*	*7*.*45*
	*SD*	*35*.*31*	*7*.*88*	*25*.*0*	*4*.*83*	*22*.*4*	*1*.*30*	*0*.*13*	*0*.*030*	*0*.*080*	*0*.*23*
cheese	18	890.0	646	622.8	52.6	568	0.74	0.09	<0.09	2.68	<2.00
cheese	19	2886	3043	2480	123	980	1.35	0.13	0.42	19.3	10.9
	*Mean*	*1888*	*1844*	*1552*	*87*.*8*	*774*	*1*.*05*	*0*.*11*	*0*.*25*	*11*.*0*	*6*.*46*
	*SD*	*1411*	*1695*	*1313*	*49*.*8*	*291*	*0*.*43*	*0*.*030*	*0*.*23*	*11*.*8*	*6*.*31*
apple	20	277.0	<92.5	49.9	29.7	30.8	2.52	0.14	0.36	0.20	3.24
pear	21	843.0	<92.5	80.8	32.1	25.1	1.18	0.27	0.59	0.48	3.61
banana	22	1972	<92.5	116	126	51.5	1.87	0.75	0.46	1.36	11.9
	*Mean*	*1031*	*<92*.*5*	*82*.*1*	*62*.*6*	*35*.*8*	*1*.*86*	*0*.*39*	*0*.*47*	*0*.*68*	*6*.*24*
	*SD*	*863*.*0*	*-*	*33*.*0*	*54*.*8*	*13*.*9*	*0*.*67*	*0*.*32*	*0*.*11*	*0*.*61*	*4*.*89*
*Cereal cream*	23	895.0	<500	251	1042	757	3.40	9.40	1.2	11.7	25.0
	24	500.0	<500	111	376	452	2.50	1.00	<0.5	2.70	14.0
	25	3635	<500	329	1467	1050	12.20	6.90	2.4	10.8	15.0
	*Mean*	*1677*	*<500*	*230*	*962*	*753*	*7*.*80*	*5*.*77*	*1*.*37*	*8*.*40*	*18*.*6*
	*SD*	*1707*	*0*	*110*	*550*	*299*	*5*.*36*	*4*.*31*	*0*.*96*	*4*.*96*	*6*.*08*

For mean calculation, when element concentration fell below the LOD, the LOD value was considered. Among essential elements (Table 3), Mg, K, P, S, Fe, Zn, and Mn were detectable in 100% of samples, Cu in 92.0%, Si in 72.0%, and Ca in 68.0%. Notably, the concentrations of essential elements in powdered milk align reliably with those reported on the labels by the manufacturers (Table 1).

Comparison of mean concentration among different types of food (Table 3) reveals that powdered milk exhibited the highest concentration for K, Ca, P, S, Fe, Cu, and Zn. Cereal cream showed the highest concentration for Si and Mn, while cheese showed the highest for Mg.

The values obtained in this study are in good agreement with those in the literature pertaining to the same food category (Table 4). Among the toxic elements (Table 5), Sb and Hg were detectable in 100% of samples; Sr in 96%, Sn in 68%; Al 36%, Ni in 16%, and As in 12%.

**Table 4:** Comparison of the max and mean concentration (mg kg^-1^) of essential (major and minor or trace) elements in baby food, between the present work and some literature values.

Country (reference)	Sample class (numbers)	K	Ca	P	Zn	Cu	Fe	Mn
Italy (present study)	Powdered milk (10)	7403–5851	4597–3870	2999–2441	68.3–47.1	3.6–3.1	53.7–46.5	1.2–1.0
	Baby food^(^^a^^,^^b^^,^^c^^,^^d^^)^ (15)	3635–1031	100.7-<92.5	1467–62.6	13.7–0.68	2.4–0.22	12.2–1.86	9.40–0.13
EU [34]	Infant formula (30)				2.9–16.5		21–27	1.5–3.3
Norway [22]	Infant formula (2)		3380±757		33.1±5.3	33.0±8.5	51±11	
Norway [22]	Fruit purèe		103±7.5		1.1±0.7	0.53±0.16	1.9±1.0	
Spain [35]	Baby food^(^^a^^,^^b^^,^^c^^)^ (23)		1670–160		8.36–1.81	1.78–0.23	65.8–2.9	13.2–0.81
UK [36]	Baby food^(^^a^^,^^d^^)^ (9)	1400–1260	564–17.4		5.4–3.4	0.5-<0.06	8.0–5.0-	

^(a)^meat

^(b)^fish

^(c)^cereals

^(d)^vegetables

**Table 5:** Concentration (mg kg-1ww) of non essential or toxic elements in baby food, minimum, maximum value, mean, standard deviation (SD).

Food	Number	Al	As	Hg	Ni	Sb	Sn	Sr
*Powdered milk*	1	< 2.5	< 0.1	0.039	< 0.5	0.200	< 0.5	1.80
	2	< 2.5	< 0.1	0.037	< 0.5	0.100	< 0.5	1.50
	3	< 2.5	< 0.1	0.031	< 0.5	0.100	< 0.5	2.20
	4	< 2.5	< 0.1	0.023	< 0.5	0.100	< 0.5	2.40
	5	< 2.5	< 0.1	0.024	< 0.5	0.100	< 0.5	1.70
	6	< 2.5	< 0.1	0.050	< 0.5	0.100	< 0.5	1.50
	7	< 2.5	< 0.1	0.016	1.9	0.200	< 0.5	2.00
	8	< 2.5	< 0.1	0.047	< 0.5	0.100	< 0.5	2.20
	9	< 2.5	< 0.1	0.028	< 0.5	0.100	< 0.5	1.40
	10	< 2.5	< 0.1	0.023	< 0.5	0.100	< 0.5	1.90
	*Mean*	*< 2*.*5*	*< 0*.*1*	*0*.*032*	*0*.*64*	*0*.*120*	*< 0*.*5*	*1*.*86*
	*SD*	*-*	*-*	*0*.*011*	*0*.*44*	*0*.*040*	*-*	*0*.*34*
*Homogenized food*								
rabbit	11	1.242	<0.018	0.0043	0.090	0.018	0.324	0.432
lamb	12	0.475	<0.019	0.0047	0.095	0.019	0.247	0.342
turkey	13	0.448	<0.016	0.0043	0.080	0.016	0.240	0.176
veal	14	0.848	<0.016	0.0027	0.080	0.016	0.256	0.288
	*Meat mean*	*0*.*753*	*<0*.*017*	*0*.*0040*	*0*.*086*	*0*.*017*	*0*.*267*	*0*.*309*
	*SD*	*0*.*370*	*-*	*0*.*0009*	*0*.*008*	*0*.*002*	*0*.*039*	*0*.*107*
bream	15	0.405	0.0750	0.0071	0.075	0.015	<0.075	0.585
salmon	16	<0.380	0.0900	0.0068	0.090	0.045	<0.075	0.540
sea bass	17	0.380	0.0150	0.0065	0.075	0.030	<0.075	0.615
	*Fish mean*	*0*.*390*	*0*.*0600*	*0*.*0068*	*0*.*080*	*0*.*030*	*<0*.*075*	*0*.*580*
	*SD*	*0*.*017*	*0*.*0397*	*0*.*0003*	*0*.*009*	*0*.*015*	*-*	*0*.*038*
cheese	18	<0.500	<0.0200	0.0022	0.100	0.020	0.320	0.540
cheese	19	<0.650	<0.0260	0.0107	0.130	0.026	0.130	2.444
	*Cheese mean*	*<0*.*580*	*<0*.*0230*	*0*.*0064*	*0*.*115*	*0*.*023*	*0*.*225*	*1*.*492*
	*SD*	*-*	*-*	*0*.*0060*	*0*.*021*	*0*.*004*	*0*.*134*	*1*.*346*
apple	20	0.684	<0.0180	0.0058	0.090	0.036	0.090	<0.306
pear	21	0.513	<0.0190	0.0086	0.190	0.018	0.095	<0.361
banana	22	<0.550	<0.0220	0.0073	0.132	0.022	0.110	<0.352
	*Fruit mean*	*0*.*580*	*<0*.*0197*	*0*.*0072*	*0*.*137*	*0*.*029*	*0*.*098*	*<0*.*340*
	*SD*	*0*.*090*	*0*.*0021*	*0*.*0014*	*0*.*050*	*0*.*010*	*0*.*010*	*0*.*030*
	*Homogenized mean*	*0*.*589*	*0*.*029*	*0*.*0059*	*0*.*102*	*0*.*023*	*0*.*170*	*0*.*582*
	*SD*	*0*.*249*	*0*.*025*	*0*.*0024*	*0*.*033*	*0*.*009*	*0*.*099*	*0*.*602*
*Cereal cream*	23	< 2.5	< 0.1	0.018	< 0.5	0.10	< 0.5	0.50
	24	< 2.5	< 0.1	0.040	< 0.5	0.10	< 0.5	<0.50
	25	< 2.5	< 0.1	0.031	< 0.5	0.10	< 0.5	2.40
	*Mean*	*<2*.*5*	*< 0*.*1*	*0*.*030*	*< 0*.*5*	*0*.*10*	*< 0*.*5*	*1*.*13*
	*SD*	*-*	*-*	*0*.*011*	*-*	*0*.*00*	*-*	*1*.*10*

Al and As were below the LOD respectively in powdered milk, cheese, cereal cream, and in powdered milk, cheese, cereal cream meat, fruit, while Sn was below the LOD in powdered milk, fish, and cereal cream. Notably, Hg, Sr, and Sb exhibited the maximum concentration in powdered milk and the minimum concentration in meat.

The comparison between mean concentrations of Sr and Sb in different food items indicates that Sr concentration was generally ten to twenty times higher than that of Sb and one hundred times higher than that of Hg.

**Fig 1:**
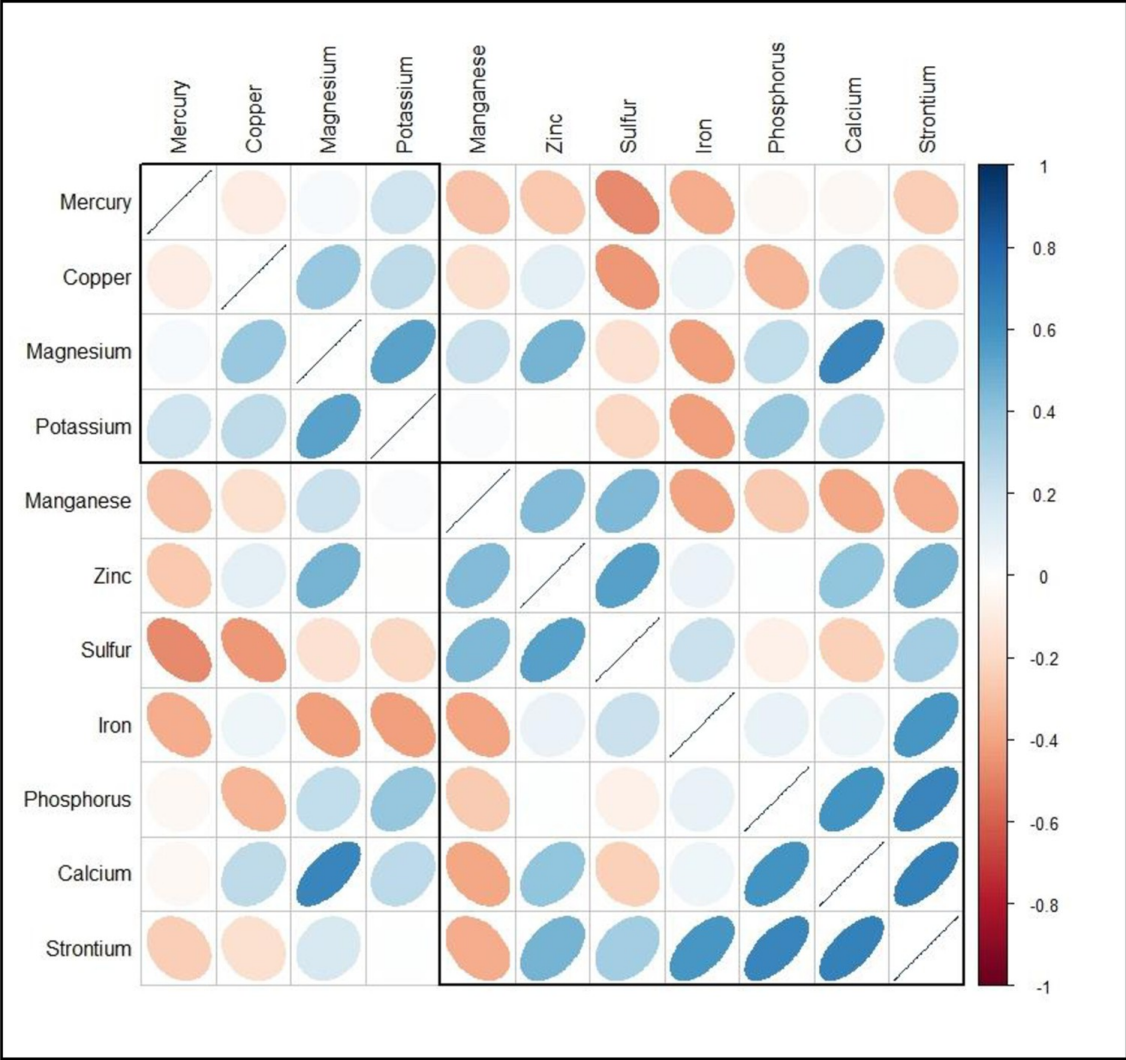
Correlation for essential (macro and trace) and toxic elements determined in samples of powdered milk.

Pearson coefficients (r) highlighting correlations between element concentrations are presented in Fig 1. Positive correlations were observed between Ca vs Sr (r = 0.62), indicative of their commonalities as alkaline earth elements. Similarly, strong positive correlations were found between P vs Ca (r = 0.84) and moderate correlation between P vs Sr (r = 0.45). Significant correlations (0.60 > r > 0.48) were found between Mg vs K (r = 0.60) and vs Zn (r = 0.48), as well as between Cu vs Mn (r = 0.61). Moderate correlations (r > 0.40) were found between Sr vs Fe, Sr vs Zn, and Cu vs Ca. Negative substantial correlations were observed between Sr vs Mn and Cu (-0.70 > r > -0.50), and Cu vs Ca (r = -0.53) and Cu vs P (r = -0.60). Furthermore, a moderate negative correlation was observed between Fe vs K and Mg (r > -0.50).

Different countries and committees have established varying micronutrient reference values for infancy [32]. This study utilizes the Population Reference Intakes (PRIs) and Adequate Intakes (AIs) [19] to evaluate the benefits of baby food for infants (Table 6). These values specify the quantity of a nutrient that needs to be regularly consumed to maintain health in an otherwise healthy individual or population. Table 6 illustrates the maximum and mean concentrations (mg kg^-1^), daily intake (mg day^-1^), and the comparison with nutritional requirements for infants aged 6–12 months for selected essential elements. Concerning essential macro-elements (K, Ca, and P), the consumption of baby food results in daily intake of potassium (calculated from mean concentrations) ranging from 14.3 (fish products) to 250 (powdered milk) mg day^-1^, representing 1.9–33% of the potassium AI. The daily intake of calcium (calculated from mean concentrations) ranges from 1.27 (fish products) to 165 (powdered milk) mg day^-1^, representing 0.45–59% of the calcium AI. Additionally, the daily intake of phosphorus (calculated from mean concentrations) ranges from 0.945 (fish products) to 119 (grain products) mg day^-1^, representing 0.59–65% of the phosphorus AI. Among trace elements (Fe, Zn, Cu, and Mn), the daily intake of iron (calculated from mean concentrations) ranges from 0.028 (fish products) to 1.99 (powdered milk) mg day^-1^, representing 0.25–18.1% of PRI. Zinc intake (calculated from mean concentrations) ranges from 0.021 (fish products) to 2.01 (powdered milk) mg day^-1^, representing 0.72–69% of the zinc AI. Moreover, copper intake (calculated from mean concentrations) ranges from 0.003 (fish products) to 0.13 (powdered milk) mg/day, representing 0.75–33% of the copper AI. A daily intake of Mn (calculated from the mean concentrations) ranging from 0.005 (meat products) to 0.71 (grain products) mg day^-1^ represents the 1.0–140% of AI. For all elements considered, the highest intake was observed in powdered milk and the lowest in fish products, except for Mn, which exhibited a high concentration in grain products. The data suggests that baby food marketed in Italy contributes significantly to the nutritional intake of Mn, Cu, Fe, Zn, Ca, K, and P.

**Table 6:** Essential element max and mean concentration (mg/kg), daily ingestion rate (kg/day), estimated max and mean daily intake (mg/day) from baby food consumption, comparison with daily nutritional requirements (PRI = Population reference intake and AI = adequate intake) (EFSA, 2017) (mg/day) for an infant (6–12 months, male or female, weaning phase).

	Element	K	Ca	P	Zn	Cu	Fe	Mn
Food (kg/day)	Adequate intake, AI or PRI (mg/day)	750	280	160	2.9	0.4	11	0.02–0.5
*Powdered milk*	Max-mean concentration	7403–5851	4597–3870	2999–2441	68.3–47.1	3.6–3.1	53.7–46.5	1.2–1.0
0.0427	Daily max-mean intake	316–250	196–165	128–104	2.92–2.01	0.15–0.13	2.29–1.99	0.051–0.043
*Meat product*	Max-mean concentration	1281–1129	<92.5	64–56	13.7–10.8	0.27–0.22	9.01–5.12	0.18–0.13
0.0411	Daily max-mean intake	52.7–46.4	<3.80	2.63–2.30	0.56–0.44	0.011–0.009	0.37–0.21	0.007–0.005
*Grain product*	Max-mean concentration	3635–1677	<500	1467–962	11.7–8.40	2.4–1.37	12.2–7.80	9.40–5.77
0.123	Daily max-mean intake	448–207	<61.7	181–119	1.44–1.03	0.29–0.17	1.50–0.96	1.16–0.711
*Root and fruits*	Max-mean concentration	1972–1031	<92.5	126–62.6	1.36–0.68	0.59–0.47	2.52–1.86	0.75–0.39
0.164	Daily max-mean intake	324–170	<15.2	20.7–10.3	0.22–0.11	0.097–0.077	0.41–0.31	0.12–0.064
*Fish products*	Max-mean concentration	1070–1046	100.7–92.7	75–69	1.59–1.51	0.27–0.24	3.57–2.07	0.54–0.41
0.0137	Daily max-mean intake	14.7–14.3	1.38–1.27	1.03–0.945	0.022–0.021	0.004–0.003	0.049–0.028	0.007–0.006

## Discussion

### Baby food: A good contribution of essential elements for the growth of the infant

Special attention is needed for infant formulas. According to the EFSA 2014 report [32], nutrients and substances should be added only in quantities that provide a nutritional or other benefit. Exceeding these necessary amounts or including unnecessary substances in formulas could impose a burden on the infant’s metabolism and other physiological functions, as unused or excess substances need to be eliminated. Therefore, maximum amounts should not be seen as target values but rather as upper limits that should not be surpassed. When milk is the sole introduced food and the estimated average consumption of milk powder is 130 g/day, the analyzed formulas meet the required nutritional needs and never exceed the maximum amount (mg/100 kcal) stipulated by Directive 2006/141/EC (Table 7).

**Table 7:** Powdered milk essential element daily ingestion rate (kg/day), max and mean concentration (mg/kg), estimate max and mean daily intake (mg/100 kcal) compared with the compositional requirements of infant formulae by Directive 2006/141/EC (EFSA, 2014).

*Powdered milk* 0.130 (kg/day)	Element	K	Ca	P	Zn		Cu	Fe	Mn
	Directive 2006/141/EC mg/100 kcal	160–60	140–50	90–25	1.5–0.5		0.1–0.035	1.3–0.3	0.1–0.001
	Max-mean concentration	7403–5851	4597–3870	2999–2441		68.3–47.1	3.6–3.1	53.7–46.5	1.2–1.0
	mg/100 kcal	119–150	79–93	60–61		1.4–0.96	0.063–0.073	1.1–0.94	0.024–0.020

### Potential health hazards resulting from baby food consumption

As far as heavy metals, the maximum limits for Pb and Sn permitted by EC Regulation 1881/06 [33] in infant formula and follow-on foods (0.02 and 50 mg kg^-1^ respectively) were used as references. The levels of Pb and Sn in the tested baby food samples were all below the maximum levels specified in Community legislation.

The Food and Agriculture Organization/World Health Organization (FAO/WHO) Joint Expert Committee on Food Additives has established the tolerable intake of heavy metals as Provisional Tolerable Weekly Intake (PTWI) or tolerable daily intake (TDI), representing the maximum amount of a contaminant that a person can be exposed to per week without an unacceptable risk of health effects.

WHO has established a Tolerable Daily Intake (TDI) for Ni at 11 μg kg^-1^ body weight per day [16], and for Hg [17], a Provisional Tolerable Weekly Intake (PTWI) at 4 μg kg^-1^ body weight/week; from this PTWI, a safety limit of 0.57 μg kg^-1^ body weight per day can be derived. In the case of inorganic As, WHO has established a PTWI of 15 μg kg^-1^ body weight per week [15]; this PTWI yields a safety limit of 2.14 μg kg^-1^body weight per day. For Al, a PTWI of 2000 μg kg^-1^body weight per week was set [18]; from this, a safety limit of 286 μg kg^-1^body weight per day can be derived. WHO also designated a PTWI for Sn at 14000 μg kg^-1^body weight per week [2]; consequently, a safety limit of 2000 μg kg^-1^body weight per day can be derived (Table 8).

**Table 8:** Toxic element max and mean concentration (mg kg^-1^), daily ingestion rate (kg day^-1^), estimated max and mean daily intake (EDI, μg kg^-1^ day^-1^) from food consumption for a body weight of 8.6 kg (EFSA, 2017) comparison with the Provisional Tolerable Daily Intake (PTDI, μg kg^-1^ day^-1^) derived from risk estimators (PTWI = Provisional Tolerable Weekly Intake = μg kg^-1^ week^-1^) (WHO, 1989, 2006, 2007, 2011a, 2011b).

Food Daily intake (kg day^-1^)		total As	Al	Hg	Sn	Ni
	Risk estimator, PTWI (μg kg^-1^ week^-1^)	15.0	2000	4.00	14000	77.0
	Risk estimator, PTDI (μg kg^-1^ day^-1^)	2.14 (inorg As)	286	0.57	2000	11.00
*Powdered milk*	Max-Mean concentration	<0.10	<2.5	0.047–0.032	<0.50	1.9–0.064
0.0427	Daily max-mean intake (μg kg^-1^ day^-1^)	<0.496	<12.4	0.233–0.159	<2.48	9.43–0.318
*Meat products*	Max-Mean concentration	<0.017	1.24–0.448	0.0047–0.0040	0.324–0.267	0.095–0.086
0.0411	Daily max-mean intake (μg kg^-1^ day^-1^)	<0.0812	5.93–2.14	0.0225–0.0191	1.55–1.28	0.454–0.411
*Grain products*	Max-Mean concentration	<0.10	<2.5	0.040–0.030	<0.50	<0.500
0.123	Daily max-mean intake (μg kg^-1^ day^-1^)	<1.43	<35.8	0.573–0.430	<7.17	<7.17
*Root and fruits*	Max-Mean concentration	<0.0197	0.684–0.580	0.0086–0.0072	0.110–0.098	0.190–0.137
0.164	Daily max-mean intake (μg kg^-1^ day^-1^)	<0.376	13.1–11.1	0.164–0.138	2.10–1.87	3.63–2.62
*Fish products*	Max-Mean concentration	0.090–0.060	0.405–0.390	0.0071–0.0068	<0.075	0.090–0.080
0.0137	Daily max-mean intake (μg kg^-1^ day^-1^)	0.143–0.0956	0.645–0.621	0.0113–0.0108	<0.12	0.143–0.127

Sample 20, homogenized food—apple, showed the highest daily intake (EDI) of Al at 13.1 μg kg^-1^body weight per day. This intake represents only about 4.58% of the daily risk estimator derived from PTWI for Al (286 μg kg^-1^ body weight per day). Sample 24, a grain product, exhibited the highest daily intake (EDI) of Hg at 0.573 μg kg^-1^body weight per day, accounting for approximately 100% of the daily risk estimator derived from PTWI for Hg (0.57 μg kg^-1^body weight per day). Sample 16, homogenized food—salmon, demonstrated the highest daily intake (EDI) of As at 0.143 μg kg^-1^body weight per day, which represents about 6.68% of the daily risk estimator derived from PTWI for As (2.14 μg kg^-1^body weight per day). Sample 7, powdered milk, displayed the highest daily intake (EDI) of Ni at 9.43 μg kg^-1^body weight per day, accounting for approximately 85.7% of the daily risk estimator derived from PTWI for Ni (11 μg kg^-1^body weight per day). Lastly, sample 22, homogenized food—banana, presented the highest daily intake (EDI) of Sn at 2.10 μg kg^-1^body weight per day, representing only about 0.03% of the daily risk estimator derived from PTWI for Sn (7000 μg kg^-1^body weight per day).

With regard to Hg, the relevant intake exceeds the daily risk estimator (PTDI) only in the consumption of the sample with the maximum concentration (sample 24, cereal cream). When considering the mean levels of Hg, its intake consistently remains below the daily risk estimator, ranging from 1.89 to 75.4% of the PTDI. Regarding arsenic, it’s essential to note that the As measured was total As and not specifically inorganic As. Estimations shown may therefore be overestimated, assuming the presence of inorganic As among the As species found in baby food.

Therefore, although the baby food commercialized in Italy is not entirely free from contaminants, heavy metal intake from baby food remains well below the recommended levels. From this perspective, the consumption of these baby products is not considered detrimental to human health.

Despite the strong recommendation for breastfeeding, the majority of infants receive infant formulae or solid foods during the first year. Ensuring the safety and suitability of foodstuffs, in terms of both the absence of contaminants and adequate micronutrient intake, is increasingly imperative for national and international health organizations, as well as for the general population. In this study, a total of 30 elements (essential and non-essential or toxic) were determined using ICP-AES and ICP-MS in 25 popular baby food products consumed in Italy and produced in Europe. Based on the data obtained, it can be concluded that the industrial baby foods investigated were not entirely free of toxic metals. However, none of the products contained concentrations of these toxic elements that could pose a health hazard to infants. The results from this survey on elemental contents and dietary infant intake complement previous research conducted by the authors on human diet and can serve as a foundation for further studies in health risk assessment.

## Conclusions

Despite the strong advocacy for breastfeeding, the vast majority of infants consume infant formulae or solid foods during their first year [2]. Ensuring the safety and suitability of these food products, in terms of both the absence of contaminants and the presence of adequate micronutrient levels, is an increasingly urgent need not only for national and international health organizations but also for the general population. In this study, a total of 30 elements (essential and non-essential or toxic) were analyzed using ICP-AES and ICP-MS in 25 popular baby food products consumed in Italy and produced in Europe. Based on the data collected, it can be asserted that the industrial baby foods investigated were not entirely devoid of toxic metals. However, none of the products contained concentrations of these toxic elements that could pose a health hazard for infants. The findings from this survey, which focus on elemental contents and dietary infant intake, complement previous research conducted by the authors on human diet and may prove valuable for further studies in health risk assessment.

## Supporting information

S1 Dataset (XLS)
